# The HAVEN study—hydroxychloroquine in ANCA vasculitis evaluation—a multicentre, randomised, double-blind, placebo-controlled trial: study protocol and statistical analysis plan

**DOI:** 10.1186/s13063-023-07108-3

**Published:** 2023-04-06

**Authors:** Annastazia E. Learoyd, Lauren Arnold, Fiona Reid, Nicholas Beckley-Hoelscher, Alina Casian, Shirish Sangle, Neil Morton, Louise Nel, Angela Cape, Susan John, Sangmi Kim, Dharshene Shivapatham, Raashid Luqmani, David Jayne, James Galloway, Abdel Douiri, David D’Cruz, on behalf of the HAVEN study group

**Affiliations:** 1 School of Life Course and Population Sciences, Faculty of Life Sciences & Medicine, King’s College London, London, UK; 2 Clinical Trial Management Platform, Guy’s and St Thomas’ NHS Foundation Trust, London, UK; 3 Louise Coote Lupus Unit, Rheumatology Department, Guy’s and St Thomas’ NHS Foundation Trust, London, UK; 4 King’s Clinical Trial Unit, Research Management and Innovation Directorate, King’s College London, London, UK; 5 Department of Immunology, School of Immunology & Microbial Sciences, King’s College London, London, UK; 6 Nuffield Department of Orthopaedics, Rheumatology and Musculoskeletal Science, University of Oxford, Oxford, UK; 7 Department of Medicine, University of Cambridge, Cambridge, UK; 8 Centre for Rheumatic Disease, Kings College London, London, UK

**Keywords:** Hydroxychloroquine, AAV, Auto-immune conditions, ANCA, Vasculitis, Prednisolone

## Abstract

**Background:**

Patients with non-severe ANCA-associated vasculitis (AAV) are often prescribed immunosuppressive medications that are associated with severe side effects and a reduced quality of life. There is an unmet need for safer effective treatments for these patients. Hydroxychloroquine is being explored due to its effect in similar autoimmune conditions such as systemic lupus erythematosus.

**Methods:**

Double-blind, placebo-controlled multicentre trial recruiting 76 patients across 20 sites. Participants will be randomised 1:1 to hydroxychloroquine or placebo in addition to standard of care immunosuppressive therapies over the course of 52 weeks. A phase II selection design will be used to determine hdroxychloroquine’s efficacy, using prednisolone dosage and Birmingham Vasculitis Activity Score as a measure of disease activity. Secondary outcomes will explore other elements of AAV progression, including disease flares and time to remission.

**Discussion:**

This trial aims to explore Hydroxychloroquine as a treatment for patients with AAV. If effective, the need for immunosuppressive treatments such as prednisolone could be reduced. Hydroxychloroquine is safer, cheaper and has fewer adverse effects than conventional immunosuppressive treatments. This could improve patient outcomes while saving money for the NHS.

**Trial registration:**

ISRCTN: ISRCTN79334891. Registered 07 June 2021.

EudraCT: 2018-001268-40. Registered 13 September 2019.

Clinicaltrials.gov: NCT04316494. Registered 20 March 2020.

**Supplementary Information:**

The online version contains supplementary material available at 10.1186/s13063-023-07108-3.

## Administrative information

**Table:** 

Title	Hydroxychloroquine in ANCA Vasculitis Evaluation - A Multicentre, Randomised, Double-blind, Placebo-controlled Trial (HAVEN)
Trial registration	ISRCTN: ISRCTN79334891 EudraCT: 2018-001268-40 Clinicaltrials.gov: NCT04316494
Protocol version	6.0 dated 10/05/2022
Funding	The trial is funded by the Medical Research Council (Grant Ref: MR/R006253/1).
Author details	^1^School of Life Course and Population Sciences, Faculty of Life Sciences & Medicine. King’s College London, UK ^2^ Clinical Trial Manager Platform, Guy’s and St Thomas’ NHS Foundation Trust, UK ^3^ Louise Coote Lupus Unit, Rheumatology Department, Guy’s and St Thomas’ NHS Foundation Trust, UK ^4^ King’s Clinical Trial Unit, Research Management and Innovation Directorate, King’s College London, UK ^5^ Dept. of Immunology, School of Immunology & Microbial Sciences, King's College London, UK ^6^ Nuffield Department of Orthopaedics, Rheumatology and Musculoskeletal Science, University of Oxford, UK ^7^ Department of Medicine, University of Cambridge, UK ^8^ Centre for Rheumatic Disease, Kings College London, UK
Name and contact information for the trial sponsor	Guy’s and St. Thomas’ NHS Foundation Trust (GSTT) Sponsor Contact: Amy Holton Address: King’s Health Partners Clinical Trials Office 16th Floor Tower Wing Guy’s Hospital London SE1 9RT Telephone: 02071885732 Fax: 02071888330 Email: amy.holton@kcl.ac.uk
Role of sponsor	GSTT is responsible for ensuring that the trial respects the dignity, rights, safety and well-being of participants. Neither the trial sponsor nor funder had direct involvement in the trial design, writing of the report, nor will they be involved in the analysis, management, interpretation and publication of the data.

## Background

The systemic vasculitides encompass a group of autoimmune inflammatory diseases affecting blood vessels of all sizes and in any organ or system. They are life-threatening diseases where the body’s defence system becomes overactive, causing inflammation of small blood vessels. The term ANCA-associated vasculitis (AAV) describes a subset of primary small vessel vasculitides characterized by the presence of anti-neutrophil cytoplasmic antibodies (ANCA): granulomatosis with polyangiitis (GPA), microscopic polyangiitis (MPA) and eosinophilic granulomatosis with polyangiitis (EGPA).

AAV are serious multisystem autoimmune disorders that can affect any organ in the body and commonly involve the ear-nose-throat, lungs, kidneys, eyes and joints. Uncontrolled disease activity can lead to organ failure and death. There are approximately 20,000 AAV patients in the UK, with 1300 diagnosed annually [1].

Although immunosuppressive treatments have improved outcomes, mortality is double that of the general population [2], 20% have persistent uncontrolled disease and 50% relapse by 5 years [3], costing > £23 million/year for hospitalisations and reducing patients’ incomes by a quarter [4].

AAV patients have a 20 times higher prothrombotic tendency [5], 3 times higher cardiovascular risk [6] and are at higher risk of immunosuppression related infections and cancer [2]. However, early therapy withdrawal, especially of glucocorticoids, is associated with disease flares, lower quality of life and increased costs [7].

There is an unmet need for safe, effective therapies for non-severe AAV to reduce disease activity, prevent disease progression and damage accumulation and minimise drug toxicity. Safer medications are needed for non-life threatening ANCA vasculitis, where aggressive immune suppressive medications are not appropriate.

Hydroxychloroquine is an effective, safe and inexpensive therapy with an established track record of disease modifying effects in autoimmune rheumatic diseases such as systemic lupus erythematosus (SLE) and Sjögren’s syndrome [8–11]. Hydroxychloroquine is safer than other immunosuppressive treatments (including in pregnancy), and it is steroid sparing, allowing reductions in cumulative prednisolone doses and their adverse effects [9]. Hydroxychloroquine has been used to treat cutaneous vasculitis [12] and rheumatoid vasculitis [10] but has never been assessed in AAV [13]. The pleiotropic effects of hydroxychloroquine on cytokines, neutrophils and autoreactive T and B lymphocytes involved in the pathogenesis of AAV provide a mechanistic rationale for its potential effectiveness [13]. Hydroxychloroquine has beneficial effects on lipids, glucose levels and arterial stiffness [14]. Hydroxychloroquine has antimicrobial [15, 16], antithrombotic [17] and antineoplastic [18, 19] effects that could be useful in immunosuppressed AAV patients. Early treatment with hydroxychloroquine could potentially reduce progression to severe disease and the need for biologic therapy, such as rituximab, which is administered for refractory or relapsing AAV [20–22]. The HAVEN trial aims to investigate this by assessing whether the addition of hydroxychloroquine to background therapy improves clinical response and quality of life in patients with AAV.

## Methods

### Trial design

**Fig. 1:**
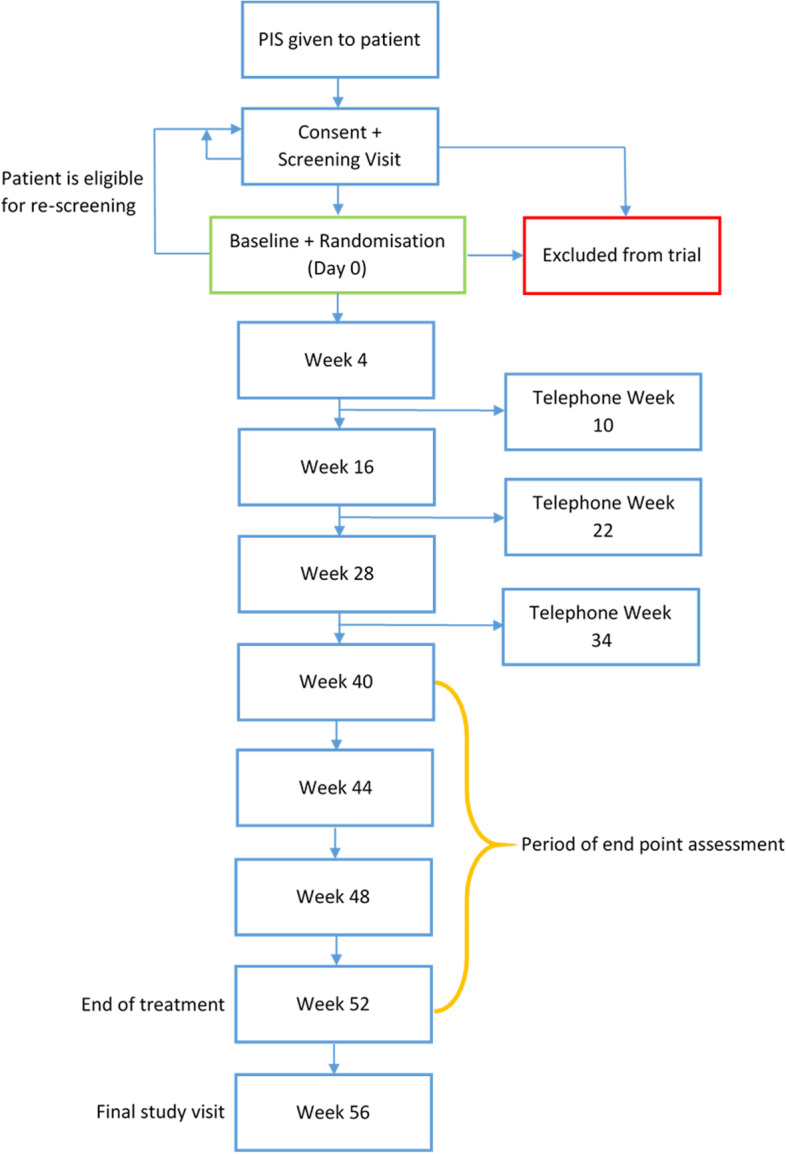
Flow chart of trial design and participant follow-up

The HAVEN trial is a double-blind, placebo-controlled multi-centre trial following a phase II selection design. Participants will be randomised to receive hydroxychloroquine or placebo in a 1:1 ratio, in addition to standard of care (SoC) maintenance therapy with prednisolone and/or stable doses of azathioprine, methotrexate, mycophenolate, co-trimoxazole or maintenance therapy with B cell depleting therapy (rituximab). Each patient will be treated with adjunctive hydroxychloroquine 400 mg daily or matching placebo for 52 weeks with dose reduction according to actual body weight and renal function. Participants will attend 10 visits across 60 weeks (Fig. 1). At each visit, disease activity will be determined using the Birmingham Vasculitis Activity Score (BVAS), and data will be collected on other measures including further disease activity indicators, treatment adherence, clinical biomarkers and quality of life (Table 1).

**Table 1:** Schedule of enrolment, interventions and assessments from SPIRIT checklist

**Study Visits**^**i**^	**Screen **	**Baseline**	**WK4**	**WK10 Telephone**	**WK16**	**WK 22 Telephone **	**WK28**	**WK 34 Telephone **	**WK40**	**WK44**	**WK48**	**WK52 End of study treatment**	**Follow up WK56**	**Withdrawal**^**ii**^
**Visit Number**	**1**	**2**	**3**	**-**	**4**	**-**	**5**	**-**	**6**	**7**	**8**	**9**	**10**	**-**
**Day (Visit window)**	**Up to 28 days**	**0**	**28** (± 7)	**70** (± 7)	**112** (± 7)	**154** (± 7)	**196** (± 7)	**238** (± 7)	**280** (± 7)	**308** (± 7)	**336** (± 7)	**364** (± 7)	**392** (± 7)	
Patient information and informed consent	X													
**Interventions:**														
IMP dispensing		X	X		X		X		X					
IMP dose recording / review of patient diary			X		X		X		X	X	X	X		X
Prednisolone dose recording	X	X	X		X		X		X	X	X	X	X	X
**Assessments:**														
COVID symptom assessment	X	X	X		X		X		X	X	X	X	X	X
Eye screen												X^iii^		
Eligibility	X	X^iv^												
Randomisation		X												
Medical history, Demographics	X													
Physical exam, Weight, Vital signs^v^, Urine dipstick	X	X	X		X		X		X	X	X	X	X	X
ECG	X				X									
Medications	X	X	X	X	X	X	X	X	X	X	X	X	X	X
BVAS^vi^	X	X	X		X		X		X	X	X	X	X	X
VDI		X			X		X			X		X		
GTI		X					X					X		
Physician’s Global Assessment	X	X	X		X		X		X	X	X	X	X	X
Urine drug screen	X													
SF-36, EQ5D, HAQ, AAV Pro		X		Xvii	X	X^vii^	X	X^vii^		X		X	X	X
FACIT score		X			X		X			X		X	X	X
Adverse event reporting		X	X	X	X	X	X	X	X	X	X	X	X	X
**Clinical Labs:**														
ANCA	X				X		X		X			X	X	X
ESR^viii^, CRP		X			X		X		X			X	X	X
Glucose		X			X		X		X			X	X	X
HbA1C	X						X					X		
Lipids	X						X					X		
Renal profile including creatinine and eGFR^ix^	X	X	X		X		X		X			X	X	X
Full blood count	X	X^x^	X		X		X		X			X	X	X
Liver function tests	X	X^x^	X		X		X		X			X	X	X
Protein: creatinine ratio^xi^	X	X	X		X		X		X	X	X	X	X	X
Viral screen^xii^	X													
Pregnancy test^xiii^	X	X												
**Research Blood Specimens:**														
Hydroxychloroquine levels		X			X		X					X		
Plasma, Serum and Cells^xiv^		X			X		X		X			X		X^xv^

^i^ Patients will be followed up by telephone at weeks 10, 22 and 34 rather than visiting the study centre. The phone call will be structured using the AAV pro questionnaire. See section 6.13 for more details

^ii^ To be arranged if a patient withdraws from the trial but is willing to have a final study visit. This is only necessary in instances where the patient’s last visit was more than 4 weeks ago

^iii^ The eye screen is only required for patients whose eGFR drops below 60ml/min at any point during the trial. The eye screen should take place between week 52 and week 56 visits by a local optometrist

^iv^ The eligibility criteria require BVAS to be scored and for female patients to have a pregnancy test at baseline as well as screening. Other screening procedures do not need to be repeated

^v^ Vital signs to include BP, pulse, respiration rate and temperature

^vi^ BVAS and VDI to be scored locally following training. BVAS scores will be quality checked by a central adjudication panel to ensure consistency

^vii^ Telephone follow up interviews will only use the AAV Pro questionnaire to structure the conversation. The SF-36, EQ5D and HAQ questionnaires will not be included

^viii^ Where ESR is not available, plasma viscosity may be used instead

^ix^ If the patient’s eGFR drops below 60ml/min at any point of the trial they should be invited for an eye screen between week 52 and 56

^x^ If the baseline visit is less than two weeks after screening, these tests do not need to be performed again

^xi^ Protein:creatinine ratio only needs to be performed if urine dipstick for protein shows 1+ or more, but results can be collected if routinely performed when the urine dipstick is negative

^xii^ Patients who test positive for hepatitis B surface antigen, hepatitis B core antibody, hepatitis C antibody or HIV-1 will be excluded

^xiii^ Urine pregnancy test

^xiv^ Plasma and serum collected at all sites. Cells at Guy’s Hospital only

^xv^ Samples only collected if the patient is identified as having a flare

### Setting

Patients will be recruited from approximately 20 NHS Trusts across the UK, including teaching and non-teaching hospital settings across England, Scotland and Wales. Patients will be identified and approached by members of their direct care team and will be given as much time as they need to make a decision about participation. Informed consent will be confirmed to the site’s principal investigator (PI) or a delegated sub-investigator. Patients who meet the eligibility criteria will be randomised.

Ten sites were initially approached to join the trial but this was expanded to 20 in September 2021 in order to address delays faced as a result of the COVID-19 pandemic. This expansion has allowed the trial to reach a larger pool of AAV patients. Sites with larger vasculitis clinics will be expected to recruit between 4 and 10 patients, with smaller community sites recruiting between 2 and 5 patients.

### Participants

Patients with ANCA-associated vasculitis (AAV) attending NHS vasculitis clinics will be approached by their direct care team to take part in the trial. Once the patient has provided consent, they will be screened against the following criteria:

#### Inclusion criteria

1. Are at least 18 years of age at screening
2. Have a clinical diagnosis of granulomatosis polyangiitis (GPA) or a diagnosis of microscopic polyangiitis (MPA) or a diagnosis of eosinophilic granulomatosis with polyangiitis (EGPA) according to the Chapel Hill Consensus Conference Definitions
3. Have a Birmingham Vasculitis Activity Score (version 3) [23] > 3 with minor BVAS items only (no major BVAS items). BVAS should be > 3 at screening and at randomisation
4. Patients should be receiving maintenance therapy at a stable dose for 4 weeks prior to randomisation. Maintenance therapy is defined as prednisolone and/or azathioprine, methotrexate, mycophenolate, co-trimoxazole or maintenance rituximab therapy
5. Patients receiving corticosteroids for reasons other than vasculitis must be on a stable regimen for 4 weeks prior to randomisation
6. A female patient is eligible to enter the study if she is:
  Not pregnant or nursing
  Of non-childbearing potential (i.e. women who have had a hysterectomy, are postmenopausal, defined as ≥ 1 year without menses, have both ovaries surgically removed or have documented tubal ligation or other permanent sterilisation procedure); or
  Of childbearing potential. These women must have a negative urine pregnancy test at screening and at baseline and be using at least one effective method of contraception

Periodic abstinence (e.g. calendar, ovulation, symptothermal, post-ovulation methods) and withdrawal are not acceptable methods of contraception. Consistent and correct use of one of the following acceptable methods of birth control for 1 month prior to the start of the study agent, during the study and 16 weeks after the last dose of study agent:

- Oral contraceptive, either combined or progestogen alone
- Injectable progestogen
- Implants of levonorgestrel or etonogestrel
- Estrogenic vaginal ring
- Percutaneous contraceptive patches
- Intrauterine device (IUD) or intrauterine system (IUS) with < 1% failure rate as stated in the product label

7. No contraindications to hydroxychloroquine therapy
8. Willing and able to give written informed consent to participate in the trial
9. Patients should have sufficient English in order to provide informed consent and complete the patient questionnaires

#### Exclusion criteria

1. Patients currently taking hydroxychloroquine or related antimalarial such as mepacrine or chloroquine
2. Patients with estimated glomerular filtration rate (eGFR) < 30 ml/min
3. Patients weighing < 40 kg
4. Sensitivity, anaphylaxis or allergy to hydroxychloroquine or any other 4-aminoquinoline compound
5. Known glucose 6 phosphate dehydrogenase deficiency
6. Known lactose intolerance
7. Evidence of plaque psoriasis
8. Concomitant use of the following medications within the last 6 months:
  Tumour necrosis factor inhibitor treatment (e.g. etanercept)
  Cyclophosphamide
  Abatacept
  Alemtuzumab
  Any experimental or biological therapies
  Intravenous, intramuscular or sub-cutaneous immunoglobulin
  Plasma exchange
  Antithymocyte globulin
  Tamoxifen
  Live vaccines
9. B cell depleting therapy (rituximab) for remission induction within the last 6 months. Rituximab maintenance therapy is permitted
10. Severe or rapidly progressive ANCA vasculitis with at least one major BVAS item
11. Have clinical evidence of significant unstable or uncontrolled acute or chronic diseases not due to vasculitis (i.e. cardiovascular, pulmonary, hematologic, gastrointestinal, hepatic, renal, neurological, malignancy or infectious disease) which, in the opinion of the principal investigator, could confound the results of the study or put the patient at undue risk
12. Patients taking long-term macrolide antibiotics for a chronic condition. This does not include topical preparations
13. Have a history of malignant neoplasm within the last 5 years, except for adequately treated cancers of the skin (basal or squamous cell) or carcinoma in situ of the uterine cervix
14. Have current drug or alcohol abuse or dependence or a history of drug or alcohol abuse or dependence within 364 days prior to randomisation. A urine drug screen should be performed and confirmed negative prior to study entry
15. Have active hepatitis B, hepatitis C or HIV-1. Patients with positive hepatitis B core antibodies may be eligible providing the viral load is undetectable and/or the patient is receiving prophylactic antiviral agents
16. Have a grade 3 or greater laboratory abnormality based on the Common Terminology Criteria for Adverse Events (CTCAE) toxicity scale (version 5), unless considered by the investigator to be related to the underlying disease or induction therapy
17. Screening 12-lead electrocardiogram (ECG) that demonstrates clinically relevant abnormalities that may affect patient safety or interpretation of study results, including: QT interval corrected using the same consistent formula at each visit (QTc) > 470 msec for female > 450 msec for male patients demonstrated by at least two ECGs
18. Participation in any other interventional trial within the last 6 months
19. Have a current symptomatic COVID-19 infection
20. Have been admitted to the ICU in the past 6 months due to a COVID-19 infection

At trial entry, eligible participants will have uncontrolled disease, which is defined as BVAS > 3. Over the course of the study, a participant may have a limited or severe flare, persistent disease, or they may enter remission. A limited flare is defined as a new or worsening minor item on the BVAS with no new major items, whereas a severe flare includes a new or worsening major item. Persistent disease indicates the presence of one or more ongoing items on the BVAS with no new or worsening items, while remission is defined as a BVAS of 0 on two consecutive visits planned to be at least 4 weeks apart.

### Intervention

Participants will be blinded and receive either 400mg daily hydroxychloroquine or placebo tablets taken orally in addition to standard of care therapies. The dose will be reduced to 200 mg daily for the duration of the trial for patients who weigh < 50 kg or who have an eGFR of 30–50 ml/min.

After study treatment, participants whose eGFR dropped below 60 ml/min at any point of the trial will be asked to attend an optometrist appointment to assess near visual acuity and visual fields.

Participants will be withdrawn from the investigational medicinal product (IMP) if they develop end stage renal failure, require a critical care admission, or if they are admitted to hospital with a COVID-19 infection. The patient may resume treatment once recovered if admitted due to COVID-19. The PI may also discontinue treatment in the event of an adverse event (AE) in accordance with best medical judgement and patients may ask to withdraw from the trial at any time.

Participants will be asked to return any unused IMP and/or empty packaging at each trial visit. The study drug returns will be returned to pharmacy for accountability. Participants will also be prompted to complete a weekly diary to record compliance. If compliance is ≤ 80%, the investigator should counsel the patient and ensure steps are taken to improve compliance. Any instances of a patient with > 120% compliance (considered an overdose) will be escalated to the trial management group (TMG) and the trial steering committee (TSC) in addition to the patient receiving counselling. Secondary levels of monitoring are provided by the DMC as part of interim data reviews and blood hydroxychloroquine levels.

Blood samples will be collected at four visits across the trial in order to analyse the levels of hydroxychloroquine in the blood. This analysis will be completed after the trial has ended to maintain blinding to treatment allocation. Samples will also be collected and stored for future research into AAV.

### Randomisation and blinding

Participants will be randomised 1:1 to the intervention or placebo with minimisation for ANCA (positive vs negative), age (</≥ 60 years), previous rituximab therapy (yes/no) and smoking (current smoker/non-smoker) to ensure balanced groups. Authorised staff members at each trial site will recruit participants and enter their details into a web-based randomisation system hosted by King’s Clinical Trial Unit (KCTU) to obtain treatment allocation. Blinded allocation details will be sent by email automatically to key trial site staff.

Both participants and research staff will be blinded to treatment allocation. Only the data monitoring committee (DMC) will have access to unblinded data during the trial period to safeguard patient interests. Blinded treatment supplies with unique treatment pack identifiers will be allocated through the King’s Clinical Trials Unit Intervention Management System. Evidence of unblinding of research staff completing data collection will be investigated.

Emergency code breaking services will be provided by ESMS Global with services available 24/7. Direct care teams and clinical trial personnel are permitted to receive unblinding information in the instance that the medical care of the patient is dependent on knowing the treatment allocation. Where possible, the PI will be contacted to confirm whether unblinding is necessary.

### Outcome measures

The primary outcome is the proportion of patients at any point during the final 12 weeks of the study (± 7 days) with uncontrolled AAV disease activity (defined as BVAS > 3) OR with controlled AAV disease activity (BVAS ≤ 3) but prednisolone dose for AAV > 7.5 mg daily OR with controlled AAV disease activity (BVAS ≤ 3) but any corticosteroid use > 7.5mg daily for any reason*.

The following secondary outcomes will be examined:

- Median cumulative number of visits where BVAS = 0 experienced by each patient (excluding screening, baseline and week 56)
- Proportion of patients with deemed treatment failure at week 52
- Mean cumulative dosage of prednisolone*
- Total number of adverse events per treatment arm*
- Median cumulative number of infections experienced by each patient
- Median number of vasculitis flares (major and minor) experienced by each patient (excluding those as screening, baseline and week 56)
- Median time from randomisation to remission (BVAS = 0 on two consecutive visits at least 4 weeks apart)
- Median time to the first severe flare with starting time points of randomisation and remission (if achieved)
- Median time to the first limited flare with starting time points of randomisation and remission (if achieved)
- Proportion of patients categorised as having a severe flare at each time point (excluding screening, baseline and week 56)
- Proportion of patients categorised as having a limited flare at each time point (excluding screening, baseline and week 56)
- Median absolute Vasculitis Damage Index (VDI) [24] at each time point and the median relative change in VDI from baseline to each time point

The following exploratory outcomes will be examined:

- Proportion of patient with incidence of new diabetes mellitus*
- Proportion of patients with dyslipidaemia
- Median Functional Assessment of Chronic Illness Therapy (FACIT) [25] score at each time point and the median relative change in FACIT score from baseline to week 52/56*
- Median scores in quality-of-life measures: SF-36 [26], EQ5D [27], Health Assessment Questionnaire (HAQ) [28] and AAV Patient Reported Outcome (PRO) [29] at each time point and the median relative change in the same quality of life measures from baseline to week 52/56*
- Median Glucocorticoid Toxicity Index (GTI) [30] at each time point and the median relative change in GTI from baseline to week 52/56
- Median Physician’s Global Assessment (PGA) [31] at each time point and the median relative change in PGA from baseline to week 52/56*
- Mean ANCA titres at each time point and the median relative change in ANCA titres from baseline to each time point*
- Proportion of patients with medicine compliance of ≤ 80%
- Mean absolute values and relative change from baseline to each time point in renal variables: serum creatinine, serum albumin, urine protein to creatinine ratio*

Participants wishing to stop trial medication will be asked to continue trial visits and data collection if willing. Otherwise, they will be withdrawn from the trial.

In the case that a participant withdraws more than 4 weeks since their last hospital visit, they will be invited to attend a withdrawal visit or a telephone interview. Data contributing to the asterisked (*) outcome measures above will be collected if feasible during this visit. Data collected prior to participant withdrawal will be included in the statistical analysis where possible.

It is anticipated that the withdrawal rate for this cohort of patients will be < 5% [21, 32, 33]. No specific plans have been made to ensure participant retention as this low withdrawal rate is based on similar studies in AAV patients. However, participants will be contacted via the telephone between hospital visits which are longer than 4 weeks apart to provide regular contact and encourage participants to report AEs.

### Sample size calculation

As AAV is uncommon, the required sample size was optimised using a phase II selection theory approach (also known as ‘pick the winner’) as proposed by Simon et al. [34]. The treatment which is ranked as having the lower proportion of patients with uncontrolled AAV (BVAS > 3) (or controlled but with > 7.5 mg prednisolone or other corticosteroid daily) in the final 12 weeks of the study will be selected as the ‘winning treatment’.

A sample size of 72 patients (36 in each arm) will be required to provide 90% probability of correctly ranking hydroxychloroquine as superior to placebo, assuming 50% of patients in the placebo group have uncontrolled AAV (or controlled but with > 7.5 mg prednisolone or other corticosteroid daily) in the final 12 weeks of the study and assuming that the true reduction in this rate due to hydroxychloroquine is 15%. This trial is not designed to demonstrate a statistically significant difference between the two arms, so the type I error rate does not apply.

Seventy-six participants will be recruited to allow for a 5% drop-out rate. This drop-out rate is based on similar trials which had a < 5% drop-out rate [21, 32, 33].

### Data management and monitoring

A web-based electronic data capture system has been designed using the InferMed Macro 4 system hosted by the King’s College London Clinical Trials Unit (KCTU). No identifiable data beyond participant initials, age at consent and year of birth will be entered on the electronic case report form (eCRF), and no data will be collected unless a participant has signed a consent form. Participants will be assigned a unique trial number upon entry to the trial, ensuring that all trial data is pseudonymised.

Source data worksheets will be used to collect data, and these will be transcribed to the eCRF. The FACIT, SF-36, EQ5D, HAQ and AAV Pro questionnaires will be used to collect quality of life data. Lab sample results will be transcribed to the eCRF from medical records.

Trial monitoring will be provided by the King’s Health Partners Clinical Trials Office (KHP-CTO). The clinical research associate (CRA) will perform source data verification and raise queries with the trial site staff. Missing data and discrepancies will be reviewed and queried by the trial’s data manager to ensure data quality prior to each extract.

#### Quality assurance

All clinicians scoring BVAS and VDI will be trained and certified prior to recruitment of participants. An adjudication committee blinded to treatment group will scrutinise all the BVAS and VDI scores and major and minor relapses. The adjudication committee will provide advice and further training where appropriate to ensure consistency across sites. The committee is chaired by Prof Raashid Luqmani, one of the sites’ principal investigators. He is the leading expert on BVAS and VDI.

#### Safety

All serious adverse events (SAEs), serious adverse reactions (SARs) and suspected unexpected serious adverse reactions (SUSARs) related to trial procedures will be reported immediately (and no later than 24 h) by the investigator to the KHP-CTO and chief investigator (CI) for review in accordance with their Pharmacovigilance Policy. Adverse events related to disease activity will be recorded as part of BVAS and so will not be reported separately. All data regarding the occurrence of adverse events will be made available to the DMC for review.

#### Trial oversight

The trial has a DMC, TSC and TMG.

The DMC meet every 6 months. The committee has access to unblinded accumulating comparative data and is comprised of independent members. The DMC is responsible for monitoring the accumulating data and making recommendations to the TSC on whether the trial should continue as planned. Only the DMC and a partially unblinded statistician has access to the unblinded accumulating data.

The TSC meet following each DMC meeting and is responsible for oversight of the trial in order to safeguard the interests of trial participants. The TSC provides advice to the CI, KHP-CTO, the funder and sponsor on all aspects of the trial through its independent chair. The BVAS adjudication committee feed into the TSC as needed.

The TMG is responsible for the day-to-day running and management of the trial and meet on a monthly basis. Sites will be invited to attend the TMG to inform them of trial amendments and provide a forum for feedback and recruitment information.

### Statistical analysis

Due to the use of a selection theory (pick the winner) approach, the main analysis will not include inferential analysis. Instead, the main analysis will involve presenting the descriptive statistics for each treatment arm to demonstrate which treatment is selected (i.e. which treatment group has a lower proportion of participants meeting the primary endpoint criteria). Due to this trial design, no interim analyses have been planned.

The descriptive statistics follow those specified in the outcome measures. The primary outcome and secondary/exploratory outcomes specifying a proportion will be described as the number and proportion of participants meeting that outcome. Secondary/exploratory outcomes specifying median will be described as median and range. Secondary/exploratory outcomes specifying mean will be described as mean and standard deviation.

#### Subgroup/exploratory analyses

The reporting of descriptive statistics for the primary endpoint and ranking of the two treatment arms will be repeated for the following subgroups:

- Those deemed compliant with treatment (consumption of > 80% of provided tablets)
- Those deemed non-compliant with treatment (consumption of ≤ 80% of provided tablets)

These analyses will only be repeated if enough participants meet these criteria for each treatment arm (≥ 5 participants per group).

Additionally, exploratory inferential analyses have been planned. This will involve tests of two proportions for those outcomes specifying a proportion. For the other outcomes, unpaired *t*-tests and Mann Whitney *U* tests will be used dependent on the resultant outcome distribution. For all tests, a one-sided significance level of 10% will be used to determine results of potential interest.

#### Considerations

Loss to follow-up is not expected as trials in a similar cohort only had a < 5% drop-out rate [21, 32, 33]. However, individual time points may experience missing data due to logistical issues. The number and proportion of participants with missing outcome data at each time point will be reported. The main analysis for this trial only considers time points in the final 12 weeks of the trial. Changes in this endpoint at each of the four visits during this period will be investigated but there is likely to be a low variability across each visit. As a result, participants with missing outcome data in one or two visits are unlikely to substantially bias the results. A sensitivity analysis will be utilised to confirm the lack of bias due to missingness in these last 12 weeks.

### Patient and public involvement

Prior to recruitment, two patients known to the CI were asked to review the trial protocol and patient information sheet. Adjustments were made to the trial documents where this was feasible. A patient representative is asked to attend each meeting of the HAVEN TSC to ensure that feedback is obtained on potential protocol amendments and issues that may arise with recruitment.

During the COVID-19 pandemic, the Vasculitis UK charity was approached to ask their members for feedback about their views on attending clinical trial visits during the pandemic. The feedback received was largely positive, but concerns about travelling on public transport were a major theme. The trial was amended to increase reimbursement for travel fees so participants could travel privately.

### Adjustments made because of COVID-19

The coronavirus pandemic delayed the trial opening and as a result the trial has been extended to cover this lost time. An amendment was made to the protocol in light of the impact of the pandemic. Firstly, the eligibility criteria was updated to exclude patients with a current symptomatic infection and those who had been admitted to critical care in the past 6 months as a result of a COVID-19 infection. Greater flexibility was added to trial visit windows to allow for participant isolation.

## Discussion

This trial aims to investigate whether the administration of hydroxychloroquine in addition to standard of care maintenance therapy can improve the clinical response of AAV by moderating disease activity, reducing disease flares and preventing disease progression, culminating in an improved quality of life for patients with this disease. The importance of developing additional treatment options for AAV cannot be understated. Although a recent meta-analysis has demonstrated a decline in mortality risk (likely due to improvements in therapeutics and patient care) [2, 35, 36], the mortality within this population is still approximately double that of the general population. Alongside this, both physical and mental quality of life for this cohort is poor compared to the general population [37], and there is little evidence that this has improved under the more recent therapeutic developments [20].

Systemic vasculitis, including AAV, is associated with increased healthcare costs due to increases in hospital care and expensive medications [4, 38, 39]. Biological therapies used for relapsing AAV cost in excess of £6000 annually per patient. On an individual level, chronic autoimmune rheumatic disorders have been associated with higher unemployment, particularly as disease duration increases [40]. AAV is unlikely to be an exception with patient reports of reduced income and hindered careers [4, 40] as a result of their disease. The increasing cost of living in the UK means that new treatments that improve patient quality of life and ability to work are greatly needed for these patients. Alongside this, the NHS has seen a decade of budget cuts, which means that inexpensive treatments would reduce the healthcare burden of these patients. Hydroxychloroquine has the benefit of being a relatively cheap medication with an estimated cost of < £70 annually per patient and it has been shown to modify disease trajectory in other related-autoimmune conditions [8–11], including SLE. Shared immunopathology between AAV and these more common autoimmune conditions suggests that Hydroxychloroquine could be just as beneficial in this patient cohort.

One key finding from studies administering hydroxychloroquine to treat rheumatoid arthritis/SLE is the potential glucocorticoid sparing effect. Glucocorticoids to induce disease remission remain the standard of care treatment for AAV, although treatment regimens have been modified over the years to minimise the cumulative dosage administered [41]. However, glucocorticoids are associated with a wide range of physical, mental and social adverse effects including the induction of long-term complications such as hypertension, diabetes, osteoporosis, cataracts and cardiovascular events [42, 43]. Similarly, the risk of infections is high, with evidence suggesting that the continued high mortality in patients may be due to treatment-related adverse events rather than AAV itself [44].

A review of off-label use of hydroxychloroquine in patients with AAV found that patients had fewer relapses, improved symptoms and a reduced need for glucocorticoids [13], and it is anticipated that these benefits will be seen in the HAVEN trial. However, a recent study by Lane et al. has shown that hydroxychloroquine increases the cardiovascular risk to rheumatoid arthritis patients after 30 days of continuous use, and this is worsened with concomitant use of azithromycin [45]. The HAVEN protocol was amended to exclude patients taking macrolide antibiotics for long-term conditions and to reintroduce an ECG at week 16 to monitor cardiovascular safety. Previous research conducted on COVID-19 patients [46] had led to this ECG being removed.

COVID-19 had a large impact on the HAVEN trial. Hydroxychloroquine received widespread media attention in the early months of the COVID-19 pandemic. Tweets sent by then-US President Donald J Trump advocating the use of hydroxychloroquine were estimated to have been seen by approximately 78 million people [47]. Awareness of hydroxychloroquine was amplified by the media’s reporting on the results of the RECOVERY trial [48] and news of the World Health Organization’s halt of the SOLIDARITY trial [49]. Concerns were raised that this news would impact recruitment to the HAVEN trial, especially given that the patient cohort was shielding, deemed to be at high risk for COVID, and reportedly avoiding medical appointments or undertaking laboratory tests [50]. The trial patient information sheet was amended to allay patient concerns about the safety of hydroxychloroquine, particularly in the light that the doses in the RECOVERY trial were 10 times greater than the doses used in HAVEN [48].

With concerns of slow recruitment, a consideration when it came to determining recruitment targets was the rarity of AAV. Approximately 1 in 3000 people within the UK population have AAV [1] meaning that this condition fits the definition for a rare disease as defined by the UK Rare Disease Framework. As a result, specific considerations were made when determining the sample size for this trial. A typical study design, with a stated type 1 error/significance level, would require at least 200 patients to compare the primary outcome in patients administered hydroxychloroquine or placebo using statistical tests with a sufficient power. For a novel therapeutic trial in a rare disease, this sample size would have been difficult to achieve, so an alternative pick the winner design was used [34]. This design describes the outcome measures in each treatment group and ranks the treatment groups but does not statistically compare them. If hydroxychloroquine ranks better than placebo (i.e. fewer patients have uncontrolled AAV or controlled AAV with high medication usage in the final 12 weeks), an extended study would be needed to accurately estimate the efficacy of hydroxychloroquine unless this effect size is substantially larger than the expected 15% used to design this trial.

Despite concerns about the impact of COVID-19 on recruitment, the HAVEN trial hopes to show that the addition of hydroxychloroquine as an adjunctive therapy to standard of care therapies does reduce disease activity in patients with AAV.

## Trial status

The trial is currently working to protocol version 6.0, dated 10 May 2022. The protocol was written in line with SPIRIT guidance.

Recruitment began on 17 December 2020 and is due to close 31 December 2024.

## Supplementary Information

**Additional file 1.**

## Data Availability

The full trial protocol is available on request to the corresponding author. The trial team are responsible for the final trial dataset. This dataset and the statistical code can be made available on reasonable request after consideration by the trial management group.

## Abbreviations

AAV: ANCA-associated vasculitis
AAV PRO: ANCA-associated vasculitis patient reported outcome
AE: Adverse event
ANCA: Anti-neutrophil cytoplasmic antibodies
BVAS: Birmingham Vasculitis Activity Score
CI: Chief investigator
CRA: Clinical research associate
CTCAE: Common Terminology Criteria for Adverse Events
DMC: Data monitoring committee
ECG: Electrocardiogram
eCRF: Electronic case report form
eGFR: Estimated glomerular filtration rate
EGPA: Eosinophilic granulomatosis with polyangiitis
FACIT: Functional assessment of chronic illness therapy
GPA: Granulomatosis with polyangiitis
GSTT: Guy’s and St Thomas’s NHS Foundation Trust
GTI: Glucocorticoid Toxicity Index
HAQ: Health Assessment Questionnaire
IMP: Investigational medicinal product
IUD: Intrauterine device
IUS: Intrauterine system
KCL: King’s College London
KCTU: King’s Clinical Trials Unit
KHP-CTO: King’s Health Partners Clinical Trials Office
MPA: Microscopic polyangiitis
PGA: Physician’s Global Assessment
PI: Principal investigator
SAE: Serious adverse event
SAR: Serious adverse reaction
SLE: Systemic lupus erythematosus
SoC: Standard of care
SUSAR: Suspected unexpected serious adverse reaction
TMG: Trial management group
TSC: Trial steering committee
VDI: Vasculitis Damage Index
