# Supplementary material for: The HAVEN study—hydroxychloroquine in ANCA vasculitis evaluation—a multicentre, randomised, double-blind, placebo-controlled trial: study protocol and statistical analysis plan
Source: Trials. 2023 Apr 6;24:261. doi: 10.1186/s13063-023-07108-3 (PMC10077754; doi:10.1186/s13063-023-07108-3)
Supplement: Supplementary file 1 — Additional file 1. [file 13063_2023_7108_MOESM1_ESM.docx]

**Hydroxychloroquine in ANCA Vasculitis Evaluation – A Multicentre, Randomised, Double-blind, Placebo-controlled Trial**

Patient Information Sheet

**We would like to invite you to take part in the HAVEN trial.**

- Before you decide whether you would like to take part in the trial, it’s important for you to understand why the research is being done and what it would involve.
- Please take some time to read the information carefully, and discuss with your family, friends and doctor, as you wish.
- If anything is not clear, or you would like some more information, please contact the study team.

**Important things that you need to know:**

- Taking part is completely up to you and you can stop taking part at any time, without giving a reason. If you do not wish to take part, this will not affect the care you receive from your doctors or other health care professionals.
- Hydroxychloroquine (HCQ) is a drug used in Rheumatoid Arthritis and Lupus, and the HAVEN study is looking at whether it is effective in patients with ANCA vasculitis.
- You will be allocated to receive either HCQ or placebo (dummy) pills by chance. You and your study doctors will not know which you receive.
- You will continue to receive your normal treatments.
- You will be asked to attend 10 visits over the course of a year. We will cover your travel expenses for these.

| **If you have any questions about this study, please contact:** |
| --- |

**What is the purpose of the study?**

Antineutrophil cytoplasmic antibody (ANCA) associated vasculitis (AAV) is a group of autoimmune diseases. This means that the immune system, which fights infections, mistakenly attacks the body’s own cells as well. In AAV, this causes blood vessels to swell or become inflamed. Treatments that suppress the immune system have been shown to improve symptoms in patients with AAV, but can cause side effects and lead to an increased risk of infection and cancer. However, stopping these treatments can be linked to flare ups of AAV and a lower quality of life. There is a need for safer and more effective treatments, particularly in patients with non-severe AAV.

This study is being conducted to see if a drug called hydroxychloroquine (HCQ) can help patients with AAV. HCQ works by reducing inflammation in people with autoimmune diseases and is used to treat conditions such as Rheumatoid Arthritis and Lupus. HCQ has been proven to be safe and effective in treating these conditions and we are hoping that using this in addition to your current therapy will better control your condition and reduce the need for high doses of steroids. Participants of this study will receive either HCQ or dummy pills (placebo) to take daily for 12 months. We are inviting 76 patients with AAV to take part.

**Why have I been approached?**

You have been invited to participate because you have been diagnosed with either Granulomatosis Polyangiitis (GPA), Microscopic Polyangiitis (MPA), or Eosinophilic Granulomatosis with Polyangiitis (also known as Churg-Strauss Syndrome).

**Do I have to take part?**

No. Taking part in the study is completely up to you. If you decide to take part you will be given this information sheet to keep and you will be asked to provide informed consent. You are free to withdraw at any time and without giving a reason. If you decide not to take part, your treatment and routine care will not be affected in any way.

**What will happen if I take part?**

Five days before every visit, we will call you to ask if you have any coronavirus symptoms. If you do, we will ask you to follow the hospital’s policy about attending your appointment.

At your first visit we will ask you to sign a consent form to confirm that you’re happy to take part in the study. The first couple of visits will include tests to check that you meet the study criteria, and that it’s safe for you to enter the study. During these visits, you will be asked to provide urine for a drug test, and female participants will also have a urine pregnancy test.

You will be asked to have an electrocardiogram (ECG) at your first and fourth visit. This is a test of your heart to make sure that HCQ is safe for you. An ECG can detect electrical signals produced by your heart, and this is done by attaching a number of sticky sensors to your skin. The ECG is a painless test but you might feel some discomfort when the sensors are removed (a bit like removing a plaster).

Patients who meet the study criteria will be placed into one of two groups. It is not possible to know beforehand which group you will be in. This is decided by chance. One group will receive HCQ tablets to take every day over the course of a year. The other group will instead receive placebo (dummy) tablets to take every day. These placebo tablets contain no active ingredients. You will have an equal chance of receiving HCQ or the dummy pills – half of the patients in the study will receive HCQ and half will receive dummy pills. You will not know which group you have been assigned to, and neither will your doctors and nurses. However, the doctors can find out if they need to. This type of study is called a randomised, double-blind, placebo-controlled trial and it ensures that the treatment is tested fairly.

You will receive either HCQ or dummy pills alongside your usual standard of care treatments. This may include steroids (prednisolone). If you are on steroids, your doctors will aim to gradually reduce your dose along the course of the study, and you will be asked to record when you take your steroids and how much you take.

You will be asked to attend 10 visits over the course of about a year. The table below provides more details about what will happen at each visit. Where possible these visits will align with your normal appointments, though there may be a need for additional visits.

|  | **Screening** | **Week 0** | **Week 4** | **Week 10 Telephone** | **Week 16** | **Week 22 Telephone** | **Week 28** | **Week 34 Telephone** | **Week 40** | **Week 44** | **Week 48** | **Week 52** | **Week 56** |
| --- | --- | --- | --- | --- | --- | --- | --- | --- | --- | --- | --- | --- | --- |
| **Visit Number** | **1** | **2** | **3** | **-** | **4** | **-** | **5** | **-** | **6** | **7** | **8** | **9** | **10** |
| Consent | x |  |  |  |  |  |  |  |  |  |  |  |  |
| Coronavirus phone call | x | x | x |  | x |  | x |  | x | x | x | x | x |
| Study drug dispensing |  | x | x |  | x |  | x |  | x |  |  |  |  |
| Steroid monitoring | x | x | x |  | x |  | x |  | x | x | x | x | x |
| Vital signs | x | x | x |  | x |  | x |  | x | x | x | x | x |
| Health assessment | x | x | x |  | x |  | x |  | x | x | x | x | x |
| Quality of Life questionnaires |  | x |  | x | x | x | x | x |  | x |  | x | x |
| Pregnancy test | x | x |  |  |  |  |  |  |  |  |  |  |  |
| Urine drug test | x |  |  |  |  |  |  |  |  |  |  |  |  |
| Clinical blood tests | x | x | x |  | x |  | x |  | x |  |  | x | x |
| Research blood tests |  | x |  |  | x |  | x |  | x |  |  | x |  |
| Electrocardiogram | x |  |  |  | x |  |  |  |  |  |  |  |  |

After you have entered the study, you will be given packs of either HCQ or placebo tablets depending on which group you are allocated to. You will also be asked to bring all of your used and unused pill packetsto each visit to help us keep track of how many pills you are taking.

We will ask you to fill in a patient diary each week to help track whether you have taken all of your tablets. It will have space for you to write whether you’ve experienced any change in your symptoms. We will ask you to bring the patient diary to every visit.

Female participants will be asked to use at least one form of effective birth control for the duration of the study until 16 weeks after the final study visit. Your doctor will discuss this with you.

With your consent we will send a letter to your GP to let them know that you are taking part in the trial.

At some of your visits we will ask you to fill in four additional questionnaires that will ask you about your quality of life. These will take you about 20 minutes to fill in. We will also ask you to provide some extra blood samples for research purposes. Where possible, these will be collected alongside routine clinical blood samples, to minimise discomfort. More details about this can be found in the next section.

Near the end of the study, once you have finished taking the study drug, we may ask you to visit your local opticians for an eye test. We will reimburse you the cost for this. In addition to the visits listed in the table we will arrange to talk to you over the telephone at a time that’s convenient for you. These calls will be arranged for weeks 10, 22 and 34. During these phone calls we will ask you how you’re feeling and if your symptoms are improving.

**Will I be asked to provide any samples?**

Routine blood tests will be performed as per your local hospital practice at each of your visits to the hospital. We will also ask for some additional blood for research purposes. These samples will be labelled by your local hospital staff using your initials and a code that can only be linked to you by the doctors at your hospital. At the end of the study, all samples taken for research will be sent to King’s College London (KCL). Your samples will not have any personal identifiable information attached to them when they are sent to KCL.

All participants will be asked to provide a total of 24mls of additional blood across four visits. This is about 1 and a half tablespoons. We are collecting this to measure the amount of the study drug found in your blood. These samples will be sent from KCL to a laboratory at Guy’s and St Thomas’ NHS Foundation Trust for analysis at the end of the study. They will destroy these samples once their analysis is completed.

**Will my samples be used in future research?**

We will ask for your consent to take blood samples for use in future research in addition to the samples mentioned above. The consent for storage of samples for future research is optional and will not affect your participation in the study in any way. If you do consent to this, we will ask you for 12mls of blood at five of your hospital visits, which is approximately 2 teaspoons per visit. We may ask you for an additional 12mls of blood if you attend the hospital for an unscheduled visit.

If you are a patient at Guy’s Hospital in London, we will ask you for a further 40mls of blood at these five visits. This is slightly more than 2 tablespoons per visit. As with the other samples collected for future research, this will not affect your participation in the current study in any way. If you do not want to provide this additional blood, but you are happy to provide the smaller amount of blood, please let the study team know.

These samples will be stored at KCL. All future research studies that involve your samples will have the approval of the Health Research Authority. None of your personal data will be shared in the event of samples being used for future research.

**What are the possible benefits and disadvantages of taking part?**

If you decide to take part in the study you will be asked to attend the hospital more frequently than you would do if you choose not to take part. Where possible, these visits will align with your usual appointments. We will reimburse you for your travel costs, up to the value of £50 per visit.

You will also receive more regular input from a study nurse and doctor to closely monitor and help you manage your vasculitis.

If you are in the group that receives HCQ, it is possible that it will help your vasculitis. However, we cannot say this for certain until we have completed this and future studies. You may not directly benefit from taking part in this study, but the information gained from your participation may help to improve the treatment of patients with your condition in the future.

There may be bruising and discomfort at the site of the blood tests, as with any blood test. However, and where possible, the blood taken for research purposes will be collected at the same time as your routine blood tests to minimise discomfort.

**What is the drug that is being tested?**

The drug we are testing is called hydroxychloroquine (HCQ). This is a safe drug used to treat patients with other autoimmune diseases, such as Lupus and Rheumatoid Arthritis.

HCQ has been used for many years and side effects are uncommon. In a small minority of people it can be associated with skin rashes, indigestion, diarrhoea, headaches and blurred vision. A small percentage of participants in previous clinical trials using similar doses of HCQ reported minor side effects. Participants taking placebo in these studies reported a similar number of minor side effects. Overall the chance of experiencing a minor side effect when taking HCQ is considered low and the chance of a serious side effect is considered to be extremely low. Your weight and kidney function will be monitored during the study and we may reduce the dose of study drug as a result.

In very rare cases, HCQ can cause visual problems. The risk of this is very small, and only affects 1% of patients after 5 years of continuous use. To minimise any risk of visual complications, we may ask you to have your vision checked at the end of the study. This can be done at your local Opticians. We will reimburse you the cost of the sight test if you provide us with a valid receipt. We will also ask you if you experience any visual problems.

**Are there any additional risks due to coronavirus (COVID-19)?**

We will take every care to make sure that you are safe if you choose to take part in the study. At every study visit to the hospital, we will give you protective face masks, gloves and an apron, if you would like. We will ring you a few days before each appointment to check whether you have any new coronavirus symptoms. If you do, you should follow the NHS guidelines for getting a coronavirus test, and let the research team know the results. If you test positive, you should stay at home in line with the hospital’s guidelines.

You may have read in the news that doctors were looking to see if HCQ could be used to treat coronavirus. One research paper wrongly suggested that coronavirus patients who were given HCQ became more unwell. This paper has been withdrawn as it was found that the results they had collected were inaccurate. Recent studies have shown that there is no advantage or disadvantage to taking HCQ for treating or preventing coronavirus. If you wish to learn more about these studies, please contact the research team.

**How will we use information about you?**

We will need to use information from you and from your medical records for this research project.

This information will include your name and contact details. People will use this information to do the research or to check your records to make sure that the research is being done properly.

People who do not need to know who you are will not be able to see your name or contact details. Your data will have a code number instead. However, we will use your initials.

We will keep all information about you safe and secure.

Once we have finished the study, we will keep some of the data for 15 years so we can check the results. We will write our reports in a way that no-one can work out that you took part in the study.

**What are your choices about how your information is used?**

- You can stop being part of the study at any time, without giving a reason, but we will keep information about you that we already have.
- If you choose to stop taking part in the study, we would like to continue collecting information about your health from your hospital. If you do not want this to happen, tell us and we will stop.
- We need to manage your records in specific ways for the research to be reliable. This means that we won’t be able to let you see or change the data we hold about you.
- If you agree to take part in this study, you will have the option to take part in future research using your data saved from this study.

**Where can you find out more about how your information is used?**

You can find out more about how we use your information:

- at [www.hra.nhs.uk/information-about-patients/](https://www.hra.nhs.uk/information-about-patients/)
- at www.guysandstthomas.nhs.uk/research/patients/use-of-data.aspx
- by asking one of the research team, or
- by sending an email to [dpo@gstt.nhs.uk](mailto:dpo@gstt.nhs.uk).

**What if relevant new information about HCQ becomes available?**

Sometimes during the course of a research project, new information becomes available about what is being studied. To ensure your safety, an independent committee of experts will review the results regularly during the study. They will also look at the results of other relevant studies. They can stop the study early if they see any unfavourable results.

If new information becomes available, your study doctor will tell you about it and discuss with you. They will explain to you any potential change to your normal care and discuss whether you want to, or should, continue in the study. If you decide to continue in the study you will be asked to sign an updated consent form.

If relevant new information becomes available your study doctor might consider it to be in your best interests for you to withdraw from the study. They will explain the reasons and arrange for your care to continue. If the study is stopped for any other reason, you will be told why, and will receive normal standard of care.

**What will happen at the end of the study?**

The study is expected to take four years to complete, starting in 2021. We are hoping to publish the results through medical publications shortly after completing the study. At this point we will be happy to send you a summarised version of the study results at your request. You will not be identifiable in the report.

Once your involvement in the study is over, you will continue to receive your usual care. At this point you may want to speak with your doctor about whether you can be prescribed HCQ.

**Who is organizing and funding the study?**

The study is funded by the Medical Research Council. The sponsor of the study is Guy’s and St Thomas’ Hospitals NHS Foundation Trust.

**Who has reviewed the study?**

This research has been reviewed by an independent group of people, called a Research Ethics Committee, to protect your safety, rights, wellbeing and dignity. This study has been reviewed and given a favourable opinion by Riverside Research Ethics Committee. The study has also been reviewed by the UK Regulatory Authority, the MHRA (the Medicines and Healthcare products Regulatory Agency). The MHRA is part of the Department of Health with the responsibility to regulate clinical trials of medicines in the UK.

**What happens if I don’t want to carry on with the study?**

You are free to withdraw your consent to participate in the study at any time and without giving a reason. This will not affect the standard of care you receive. You have the right to request samples collected as part of this study to be destroyed and no further laboratory analysis to be performed.

Your study doctor can take you out of the study at any time if it is in your best medical interests to stop your participation. The study sponsor also has the right to direct your study doctor to take you out of the study at any time.

If you have any questions that remain unanswered, your study doctor or nurse will be happy to answer these for you.

**What if something goes wrong?**

If you have a concern about any aspect of this study, you should ask to speak to the researchers who will do their best to answer your questions [insert Principal Investigator name, telephone number and e-mail address]. If you remain unhappy and wish to complain formally, you can do this through the Guy’s and St Thomas’ Patients Advice and Liaison Service (PALS) on 020 7188 8801, pals@gstt.nhs.uk. The PALS team are based in the main entrance on the ground floor at St Thomas’ Hospital and on the ground floor at Guy’s Hospital in the Tower Wing. In the event that something does go wrong and you are harmed during the research and this is due to someone‘s negligence then you may have grounds for legal action for compensation against Guy’s and St Thomas’, but you may have to pay your legal costs. The normal National Health Service complaints mechanisms will still be available to you (if appropriate).

**Thank you for taking the time to read this Patient Information Sheet.**

**Contact details**: ****PLEASE UPDATE with the site PI and site PALS details****

**Site Principal Investigator details**:

Name:

Address:

If you have any concerns about the way the study is carried out by the study staff, or your rights as a research patient, or any other aspects of your care, please contact the person below.

**Site PALS Team details**:

PALS Lead, Patient Information Team

Address:

Email:
